# An ocular Th1 immune response promotes corneal nerve damage independently of the development of corneal epitheliopathy

**DOI:** 10.1186/s12974-023-02800-2

**Published:** 2023-05-22

**Authors:** Alexia Vereertbrugghen, Manuela Pizzano, Florencia Sabbione, Irene Angelica Keitelman, Carolina Maiumi Shiromizu, Douglas Vera Aguilar, Federico Fuentes, Cintia S. de Paiva, Mirta Giordano, Analía Trevani, Jeremías G. Galletti

**Affiliations:** 1 Innate Immunity Laboratory, Institute of Experimental Medicine (CONICET/National Academy of Medicine of Buenos Aires), Buenos Aires, Argentina; 2 Confocal Microscopy Unit, Institute of Experimental Medicine (CONICET/National Academy of Medicine of Buenos Aires), Buenos Aires, Argentina; 3 Department of Ophthalmology, Ocular Surface Center, Cullen Eye Institute, Baylor College of Medicine, Houston, TX USA

**Keywords:** Corneal nerves, Neuropathy, CD4 T cells, Th1 response, Immunopathology

## Abstract

Proper sight is not possible without a smooth, transparent cornea, which is highly exposed to environmental threats. The abundant corneal nerves are interspersed with epithelial cells in the anterior corneal surface and are instrumental to corneal integrity and immunoregulation. Conversely, corneal neuropathy is commonly observed in some immune-mediated corneal disorders but not in others, and its pathogenesis is poorly understood. Here we hypothesized that the type of adaptive immune response may influence the development of corneal neuropathy. To test this, we first immunized OT-II mice with different adjuvants that favor T helper (Th)1 or Th2 responses. Both Th1-skewed mice (measured by interferon-γ production) and Th2-skewed (measured by interleukin-4 production) developed comparable ocular surface inflammation and conjunctival CD4+ T cell recruitment but no appreciable corneal epithelial changes upon repeated local antigenic challenge. Th1-skewed mice showed decreased corneal mechanical sensitivity and altered corneal nerve morphology (signs of corneal neuropathy) upon antigenic challenge. However, Th2-skewed mice also developed milder corneal neuropathy immediately after immunization and independently of ocular challenge, suggestive of adjuvant-induced neurotoxicity. All these findings were confirmed in wild-type mice. To circumvent unwanted neurotoxicity, CD4+ T cells from immunized mice were adoptively transferred to T cell-deficient mice. In this setup, only Th1-transferred mice developed corneal neuropathy upon antigenic challenge. To further delineate the contribution of each profile, CD4+ T cells were polarized in vitro to either Th1, Th2, or Th17 cells and transferred to T cell-deficient mice. Upon local antigenic challenge, all groups had commensurate conjunctival CD4+ T cell recruitment and macroscopic ocular inflammation. However, none of the groups developed corneal epithelial changes and only Th1-transferred mice showed signs of corneal neuropathy. Altogether, the data show that corneal nerves, as opposed to corneal epithelial cells, are sensitive to immune-driven damage mediated by Th1 CD4+ T cells in the absence of other pathogenic factors. These findings have potential therapeutic implications for ocular surface disorders.

## Introduction

The cornea contributes the most refractive power to the eye. To fulfill its visual function, the anterior corneal surface must remain wet, smooth, and regular despite being directly exposed to the environment [1]. The cornea is densely innervated by the ophthalmic branch of the trigeminal nerve, allowing the detection of moisture changes and potential threats [2, 3]. Because of their sensory function, corneal nerves act as guardians of the ocular surface and also serve trophic and immunoregulatory roles [4, 5]. Recently, it has become increasingly evident that corneal nerves are essential to corneal integrity, and conversely, that corneal nerve dysfunction contributes to ocular surface pathophysiology [5].

Corneal nerve damage, i.e., corneal neuropathy, has been reported in numerous ocular surface disorders: dry eye, infectious keratitis (caused by herpes viruses, Acanthamoeba, mycobacteria, and fungi), ocular graft-versus-host disease (GVHD), Stevens–Johnson syndrome, and severe forms of allergic keratitis [4–7]. All these inflammatory disorders share the fact that the underlying ocular surface immune response promotes local tissue damage [8–10]. But contrasting the abundant evidence on corneal epithelial damage mechanisms in ocular surface disease, little is known about how corneal neuropathy develops.

The distalmost portions of the corneal nerves comprise the subbasal nerves and intraepithelial nerve terminals; they are located entirely within the corneal epithelium and lack Schwann cells, thus relying solely on epithelial cells for support [11]. As the epithelium is the corneal layer most affected by inflammation in many ocular surface disorders, it is tempting to speculate that damage and dysfunction spread secondarily from the corneal epithelium to the corneal nerves as a consequence of the compromised epithelial scaffolding and nourishment. Supporting this notion, the corneal epithelial barrier in the setting of dry eye disease is disrupted by inflammation-induced metalloproteinase activity on intercellular adhesion proteins [12] and also by inflammatory cytokine-triggered epithelial apoptosis [13]. In this context of ocular surface desiccation, both Th1 and Th17 CD4^+^ T cells have been shown to drive corneal epitheliopathy [14–17]. As the development of corneal neuropathy and epitheliopathy in murine dry eye models follow a similar tempo [7], the simplest explanation would be that the intraepithelial corneal nerves suffer bystander damage as their supporting epithelial cells are affected by inflammation and desiccation.

However, in murine models of herpetic keratitis and ocular GVHD, complement activation and CD4^+^ T cells targeting disease-specific pathogenic antigens (viral proteins or allogeneic peptides) have been shown to promote corneal nerve damage [18]. Remarkably, this does not seem to be the case in an ocular allergy model, a disease that is also mediated by antigen-specific CD4^+^ T cells [18]. The contrasting findings might be explained by the different extent of corneal epithelial damage associated with each disease model or could be ascribed to the different CD4^+^ T cell responses (Th1-dominant in herpetic keratitis vs Th2-dominant in allergy) and the cytokines and effector cell types involved [13, 19–22]. Thus, herpetic keratitis, ocular GVHD, dry eye, and ocular allergy differ not only in the extent and features of the initial innate immune response, but also in the nature, location, and abundance of the ocular surface antigens targeted by the ensuing adaptive immune response [19–23]. These factors may add to the varying corneal nerve changes reported. Therefore, whether a predominant Th1, Th2, or Th17 response contributes to corneal nerve damage is unknown.

The polarization of the adaptive immune response has a profound impact on neuropathology outside the eye [24–26]. In spontaneous autoimmune and traumatic neuropathy models involving extraocular nerves, the different types of adaptive immune responses actively promote either neural damage or regeneration [27–30]. Whether this also applies to corneal neuropathy development has yet to be determined, in part due to the numerous non-immune differences between disease models discussed above. Here we hypothesized that the type of immune response at the ocular surface in and of itself contributes to corneal nerve damage independently of the disease setting. To this aim, we evaluated the effect on corneal nerves of different adaptive immune responses targeting the same antigen in otherwise comparable circumstances, thus controlling for other potential confounding factors. Our data indicate that Th1 CD4^+^ T cells are capable of inducing corneal nerve damage without causing epitheliopathy. These findings have potential implications in ocular surface therapeutics.

## Methods

### Mice

C57BL/6 (C57BL/6NCrl) mice were originally obtained from Charles River Laboratories (Wilmington, MA, USA). OT-II (B6.Cg-Tg(TcraTcrb)425Cbn/J, JAX stock #004194) and RAG1-deficient (B6.129S7-*Rag1tm1Mom*/J, JAX stock #002216) mice were purchased from The Jackson Laboratory (Bar Harbor, ME, USA). Mice were bred and maintained at the Institute of Experimental Medicine's conventional animal facility. RAG1^−/−^/OT-II mice were generated in-house by cross-breeding. All mice were 6–8 weeks old at the beginning of the experiments and both male and female mice were included. All protocols were approved by the Institute of Experimental Medicine animal ethics committee and adhered to the Association for Research in Vision and Ophthalmology Statement for the Use of Animals in Ophthalmic and Vision Research.

### Reagents, antibodies, and cell cultures

All chemical and biological reagents were from Sigma-Aldrich (Buenos Aires, Argentina) unless otherwise specified. Grade V ovalbumin (OVA) was used in all experiments. Fluorochrome-tagged antibodies were from Biolegend (San Diego, CA, USA) unless otherwise specified. All cell cultures were done in RPMI-1640 medium supplemented with 10% fetal calf serum, 10 mM glutamine, 100 U/ml penicillin, 100 μg/ml streptomycin, and 5 × 10^−5^ M 2-mercaptoethanol in a humidified incubator with 5% CO_2_ at 37 °C.

### Immunization and ocular challenge

For Th1 skewing, mice received 0.1 ml of 1:1 PBS:complete Freund’s adjuvant (CFA) emulsion containing 100 μg OVA as a subcutaneous injection in the flank [31]. For Th2 skewing, mice received 0.5 ml of a 400 μg/ml OVA + 4 mg/ml aluminum hydroxide suspension as an intraperitoneal injection [32]. For the ocular antigenic challenge, mice were instilled once daily with 5 μl/eye of 50 mg/ml OVA or saline.

### Assessment of corneal epithelial barrier function

Corneal fluorescein uptake was measured as previously described [33]. In brief, 0.5 μl of dextran-fluorescein isothiocyanate (average mol wt 3000–5000, 10 mg/ml in PBS) was applied to each eye and then the mouse was returned to its cage. After 3 min, a 10–20 s-long video of each eye under blue light was captured with the aid of a fluorescence-adapted dissection microscope (NightSea SFA-RB). For analysis, a masked observer exported a representative video frame as an image and selected the corneal area suitable for analysis, excluding reflections and other artifacts, using ImageJ software. The mean fluorescence intensity of the resulting region of interest was calculated after background subtraction (50-pixel rolling ball radius), and the average of both eyes was used for analysis.

### Assessment of corneal mechanical sensitivity

Mechanical thresholds were determined using a mouse-adapted version of Cochet-Bonnet esthesiometry [7, 34]. Nylon 6-0 monofilament was cut into segments of varying lengths (1.0 to 5.5 cm in 0.5 cm steps). With the mouse held firmly in one hand, the cornea was touched six times with each filament, starting with the longest segment. A positive response was defined as blinking and retraction of the eye in reaction to at least three of the six tries. The longest segment yielding a positive response was used as the sensitivity threshold, and the average of both eyes was used for analysis. Corneal sensitivity was measured in the morning (8–11 AM) before any other experimental manipulation.

### Delayed type-hypersensitivity (DTH) assay

Mice were anesthetized with isoflurane and then heat-aggregated OVA (100 μg in PBS) and PBS alone were injected in a volume of 30 μl into the right and left footpads, respectively. Antigen-induced swelling was measured 48 h later with a dial thickness gauge as the mean difference in thickness between the right and left footpads of each mouse [35].

### Enzyme-linked immunosorbent assay (ELISA)

OVA-specific IgE levels in serum were determined with a previously validated indirect ELISA [32]. Blood was collected by cardiac puncture immediately after euthanasia and the sera were stored at − 80 °C until assaying. For the ELISA, 96-well microtiter plates were coated overnight with 100 μg/ml OVA in PBS, blocked with 5% BSA for 2 h, incubated with 1:20 and 1:200 serum dilutions for 2 h, then incubated with 5 μg/ml purified anti-mouse IgE biotinylated antibody (cat #406904, BioLegend), and finally, horseradish peroxidase and chromogenic substrate were added at previously determined optimal concentrations. The reaction was stopped with 1 N H_2_SO_4_ and absorbance at 405 nm was measured with a reference filter set to 570 nm.

### Spleen and lymph node cells

Cervical, axillary, and inguinal lymph nodes were harvested after euthanasia and rendered into a cell suspension by mechanical dissociation through nylon mesh. For splenocyte suspensions, red blood cells were lysed with ammonium chloride–potassium buffer.

### Intracellular cytokine staining

Cells were stimulated in U-bottom 96-well plates (0.5 × 10^6^ cells/0.2 ml media/well) for 5 h with 50 ng/ml phorbol myristate acetate (PMA), 1 µg/ml ionomycin, and 10 µg/ml brefeldin A. DNAse (1 U/ml) was added 15 min before the stimulation period ended. The cells were then washed, stained with a fixable viability dye (#L34975, Thermo Fisher Scientific, Buenos Aires, Argentina), washed, stained with CD4 (CD4 FITC #100406, Biolegend), and then fixed, permeabilized, and stained for intracellular cytokines (IFN-γ PE #505808, IL-4 BV421 #504120, IL-17A PE-Cy7 #506922, Biolegend) with the Cyto-Fast™ Fix/Perm Buffer Set (#426803, BioLegend) as per the manufacturer’s instructions.

### Collection of eye tissue

After euthanasia, the conjunctival tissue of each eye was excised as two strips (superior and inferior) under a dissection microscope and collected in ice-cold RPMI media without serum. Immediately after, enucleation was performed by gently proptosing the eye globe and cutting the optic nerve with curved scissors. The two eyes of each mouse were collected in ice-cold fixative solution. Mice were euthanized one at a time so that all ocular tissue was collected within 5 min of the time of death to ensure adequate corneal nerve preservation [36].

### Corneal immunostaining and confocal laser scanning microscopy acquisition

Eyes were processed as described by Tadvalkar et al. [36]. In brief, eyes were fixed in a pre-chilled formaldehyde-containing buffer for 75 min, washed, and stored in methanol at − 20 °C until processed for staining. Then, the fixed corneas were cut from the back of the eye under a dissection microscope, permeabilized with a graded methanol–Triton X-100 series, blocked overnight with 1% BSA and 1% goat serum in PBS, and stained overnight with Alexa 488-conjugated Alexa Fluor® 488 anti-tubulin β3 and Alexa Fluor® 647 anti-mouse/human CD324 (E-cadherin) antibodies (#801203 and #147308, BioLegend). Each batch of anti-tubulin β3 antibody was titrated before use to minimize background staining, usually resulting in 0.5–0.7 μl antibody/200 μl buffer/cornea (2.5–3.5 μg/ml) as optimal. The stained corneas were washed three times for 60 min in PBS–Tween 0.02%, counterstained with 1 μg/ml DAPI, mounted flat with the aid of relaxing cuts in Aqua-Poly/Mount (PolySciences), and stored at 4 °C until imaged.

Image acquisition was performed with a FluoView FV1000 confocal microscope (Olympus, Tokyo, Japan) equipped with Plapon 60X/1.42 and UPlanSapo 20X/0.75 objectives. Z stacks (0.5-μm step size) spanning the entire corneal epithelium (approximately 30 μm) were obtained at two opposite located at 600 μm from the center (defined as the center of the nerve whorl or as the center of the disorganized area in those samples with highly disrupted nerve whorls). Corneal nerve analysis was performed at three different levels within the corneal epithelium. For subapical nerve terminals, the first section located entirely beneath the apical epithelial squamous cells (1–1.5 μm deep, usually the third or fourth) was selected. Then, the image was thresholded after background subtraction (10-pixel rolling ball radius) and the percentage area occupied by nerve endings was determined by the corresponding ImageJ function. For mid-epithelial nerve terminals, the mid-section (60 ×) between the apical- and basal-most sections from each stack was chosen. Then, the number of nerve endings was assessed after background subtraction (10-pixel rolling ball radius) by a masked observer using the Cell Counter ImageJ function. Data are shown as the number of terminals/60 × field (423.94 µm^2^ area). To analyze the complexity of the subbasal epithelial nerves, the Sholl plugin in ImageJ software was used. In brief, a maximum intensity projection of the 10 sections encompassing the corneal subbasal nerve mat was created, then the background was subtracted (50-pixel rolling ball radius), and the image was thresholded. Finally, 10 concentric circles with a 10-µm radius step size were traced at the center of the final image and the resulting sum of intersections of tubulin β3^+^ nerves for each concentric circle was calculated using the software and used for analysis [32].

### Preparation and flow cytometry analysis of conjunctival cell suspensions

Conjunctival tissue was minced into fragments with the aid of scissors, incubated in collagenase 1 mg/ml in PBS at 37 °C with gentle shaking for 30 min, then DNAse 2 U/ml was added and the tissue samples were digested for another 15 min. Digestion was stopped by adding 2 mM EDTA and 10% fetal calf serum and the suspension was washed and filtered for staining. Cell suspensions were first stained with a fixable viability dye (#L34975, Thermo Fisher Scientific, Buenos Aires, Argentina), washed, Fc-receptor blocked, stained for surface markers (CD45 APC #103112, CD4 FITC #100406, Ly6G PE-Cy7 #127618, Biolegend), and then fixed in 1% paraformaldehyde in PBS. The entire cell suspension resulting from one mouse was stained and acquired for analysis as one independent sample. First, singlets were gated based on forward scatter height versus area, then gated on side scatter height versus side scatter area, then gated on viability dye-excluding events (viable cells), and finally on CD45^+^ CD4^+^ or CD45^+^ Ly6G^+^ events.

### Isolation and adoptive transfer of CD4+ T cells

CD4^+^ cells were isolated from pooled splenocytes and lymph node cells with the aid of magnetic beads (MojoSort™ Mouse CD4^+^ T Cell Isolation Kit, BioLegend #480,033) as per the manufacturer’s instructions. Cell purity was > 90% as assessed by flow cytometry (CD4 staining). For adoptive transfer experiments, cells were resuspended in PBS and 1 × 10^6^ cells/0.5 ml were injected intraperitoneally into each RAG1^−/−^ recipient mouse.

### In vitro polarization of CD4^+^ T cells

We adapted a previously published protocol [37]. For all conditions, magnetically isolated CD4^+^ T cells from RAG1^−/−^/OT-II mice were seeded at 1 × 10^6^ cells/ml in complete medium on anti-mouse CD3ε-coated plates (clone 145-2C11, #100339, Biolegend, 1 µg/ml, overnight incubation). The following polarization cocktails were added immediately after seeding (day 0): for Th1 cultures, 3 µg/ml anti-mouse CD28 (clone 37.51, #102116, Biolegend), 10 µg/ml anti-mouse IL-4 (clone 11B11, #504122, Biolegend), 5 ng/ml recombinant mouse IL-2 (5 ng/ml, # 575402, Biolegend), and 10 ng/ml recombinant mouse IL-12 (#577002, Biolegend) were added; for Th2 cultures, 3 µg/ml anti-mouse CD28, 5 ng/ml recombinant mouse IL-2, and 50 ng/ml recombinant mouse IL-4 (#574302, Biolegend) were added; and for Th17 cultures, 3 µg/ml anti-mouse CD28, 50 ng/ml recombinant mouse IL-6 (#575,704, Biolegend), 1 ng/ml recombinant human TGF-β1 (#781,802, Biolegend), 5 ng/ml recombinant mouse IL-23 (#589002, Biolegend), 10 µg/ml anti-mouse IL-4, and 10 µg/ml anti-mouse IFN-γ (clone XMG1.2, #505834, Biolegend) were added. On day 3, 50% of the media with polarization cocktails was replaced, and on day 5, the cells were harvested, washed twice to remove any traces of cytokines and antibodies, and the resulting polarization was assessed by intracellular cytokine staining. For adoptive transfer, the cells were washed once more in PBS before adjusting the concentration for injection.

### Statistical analysis

Student’s *t*-test and one- or two-way analysis of variance (ANOVA) with Bonferroni or Dunnett’s post hoc tests were used to compare the means of two or more samples, respectively. Significance was set at *p* < 0.05 and two-tailed tests were used in all experiments. Calculations were performed using GraphPad Prism version 7 software (GraphPad Software, La Jolla, Ca, USA).

## Results

### Only a Th1-skewed immune response at the ocular surface induces corneal neuropathy in T cell receptor-transgenic mice

**Fig. 1:**
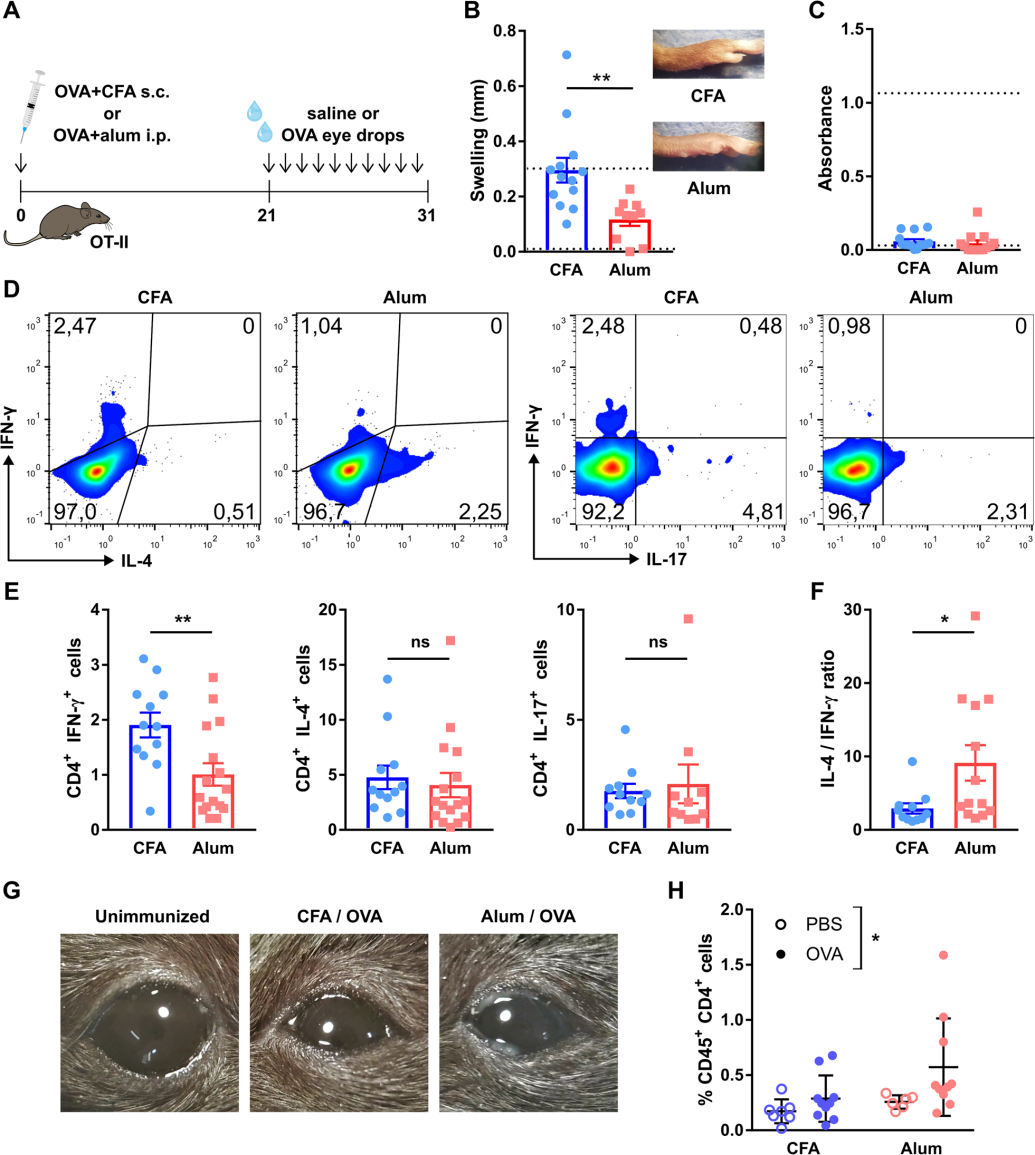
Th1 and Th2-skewing of the adaptive immune response in the ocular surface of transgenic CD4^+^ T cell receptor mice. **A** OT-II mice [transgenic for an ovalbumin (OVA)-specific MHC II-restricted T cell receptor) were immunized with OVA in combination with either complete Freund’s adjuvant (CFA) or alum to induce a Th1- or Th2-skewed immune response, respectively. Three weeks later (day 21), mice were given saline or OVA eye drops daily for 10 days to induce an ocular immune response. **B** Delayed-type hypersensitivity response to footpad OVA injection in immunized OT-II mice. The reference line corresponds to historical data from the same assay performed on wild-type C57/BL6 mice. Cumulative data (left) and representative images (right) of footpads. **C** Serum OVA-specific IgE levels in OT-II mice 30 days after immunization as assessed by ELISA (representative experiment). Upper and lower reference lines correspond to positive (alum-immunized) and negative (non-immune) wild-type controls. **D** Representative dot plots and **E** cumulative data of interferon-γ (IFN-γ), interleukin (IL)-4, and IL-17 production by CD4^+^ T cells obtained from spleens of immunized mice. **F** Cumulative data of ratio of IL-4/IFN-γ producing CD4^+^ T cells. **G** Representative photographs of mouse eyes on day 31 of the experiment (after 10 days of antigenic challenge). **H** Conjunctival CD4^+^ T cells in immunized mice on day 31 as assessed by flow cytometry (representative experiment). All experiments were performed twice or more with 6 mice/group/experiment. For all experiments, mean ± standard error of measurement is shown. To compare means, Student’s *t* test was used for **B**, **C**, **E**, and **F**, and two-way ANOVA (immunization and ocular challenge) with Sidak’s post hoc test was used for **H**. *p < 0.05, **p < 0.01, and *ns* not significant

Previous reports showed that corneal nerve damage develops in murine models of dry eye [7, 38], herpetic keratitis, and ocular GVHD, but not in ocular allergy [18]. The former three disorders have in common a Th1-predominant immune response, while the latter is Th2-predominant. There are, however, many other pathogenic differences and similarities among them, including the extent and mechanism of corneal injury and the activation of the innate immune system. Here we hypothesized that a Th1-biased ocular immune response in and of itself promotes corneal neuropathy independently of additional non-immune noxae such as desiccation, viral infection, or chemical injury. As a proof-of-concept, we initially immunized two groups of OT-II mice (transgenic for an ovalbumin (OVA)-specific MHC II-restricted T cell receptor) with the same antigen (OVA) but combined with different adjuvants that enhance and condition the adaptive immune response. For this experiment, we compared complete Freund’s adjuvant (CFA), which induces a Th1-skewed immune response, and alum, which has the opposite effect of favoring Th2-skewed immune responses (Fig. 1A) [39]. The choice of adjuvants was based on their well-described polarizing effect, which was in fact instrumental to the discovery and characterization of Th1 and Th2 immune responses [40–42].

After allowing 3 weeks for the systemic immune response to develop in response to immunization, we challenged the mice with eye drops containing either OVA or vehicle (Fig. 1A). The rationale was to locally activate the previously developed, Th1/Th2-biased immune response on an ocular surface without a preexisting innate immune response to injury. First, we corroborated the Th1 vs Th2-skewing of the resulting antigen-specific immune response. To this aim, mice were challenged with OVA in one footpad 3 weeks after immunization to elicit a delayed-type hypersensitivity response, an in vivo readout of cellular immunity that is driven mostly by antigen-specific Th1 CD4^+^ T cells [43] but not by Th2 CD4^+^ T cells [42]. Consistently, CFA-immunized mice (Th1-biased) showed strong delayed type-hypersensitivity reactions to OVA (Fig. 1B), a sign of Th1 skewing [44]. By contrast, alum-immunized mice (Th2-biased) displayed weaker responses suggestive of Th2 skewing [42]. Moreover, considering the fact that OT-II mice are Th1 prone due to their C57BL/6 background, this finding already shows that alum immunization diminished the default Th1 immune response [45]. Conversely, antigen-specific IgE production is a hallmark of Th2 immune responses because it is driven by Th2 CD4^+^ T cells and opposed by Th1 cytokines [46, 47]. However, this readout (Fig. 1C) was inconclusive in alum-immunized OT-II mice (Th2-biased) because this strain mounts defective humoral responses in general [45]. We then resorted to directly quantifying cytokine profiles by flow cytometry. Appropriate assessment of Th1/Th2-skewing requires analyzing the relative change in cytokine production due to their contraposing effects and different potency [44, 48]. Splenic CD4^+^ T cells from Th1-skewed mice produced more interferon-γ (IFN-γ), the signature Th1 cytokine, than those from Th2-skewed mice upon in vitro stimulation (Fig. 1D, E). Under the same conditions, both cell groups produced comparable levels of interleukin (IL)-4, a signature Th2 cytokine, or IL-17, the signature Th17 cytokine (Fig. 1D, E) [49]. The IL-4/IFN-γ ratio portrays the immune skewing more clearly: alum-immunized mice had a significantly higher IL-4/IFN-γ ratio of cytokine-producing cells (Fig. 1F), as expected in Th2 polarization [44, 48, 50]. Thus, cytokine profile assessment confirmed the in vivo findings of strong Th1/Th2 polarization after CFA/alum immunization.

**Fig. 2:**
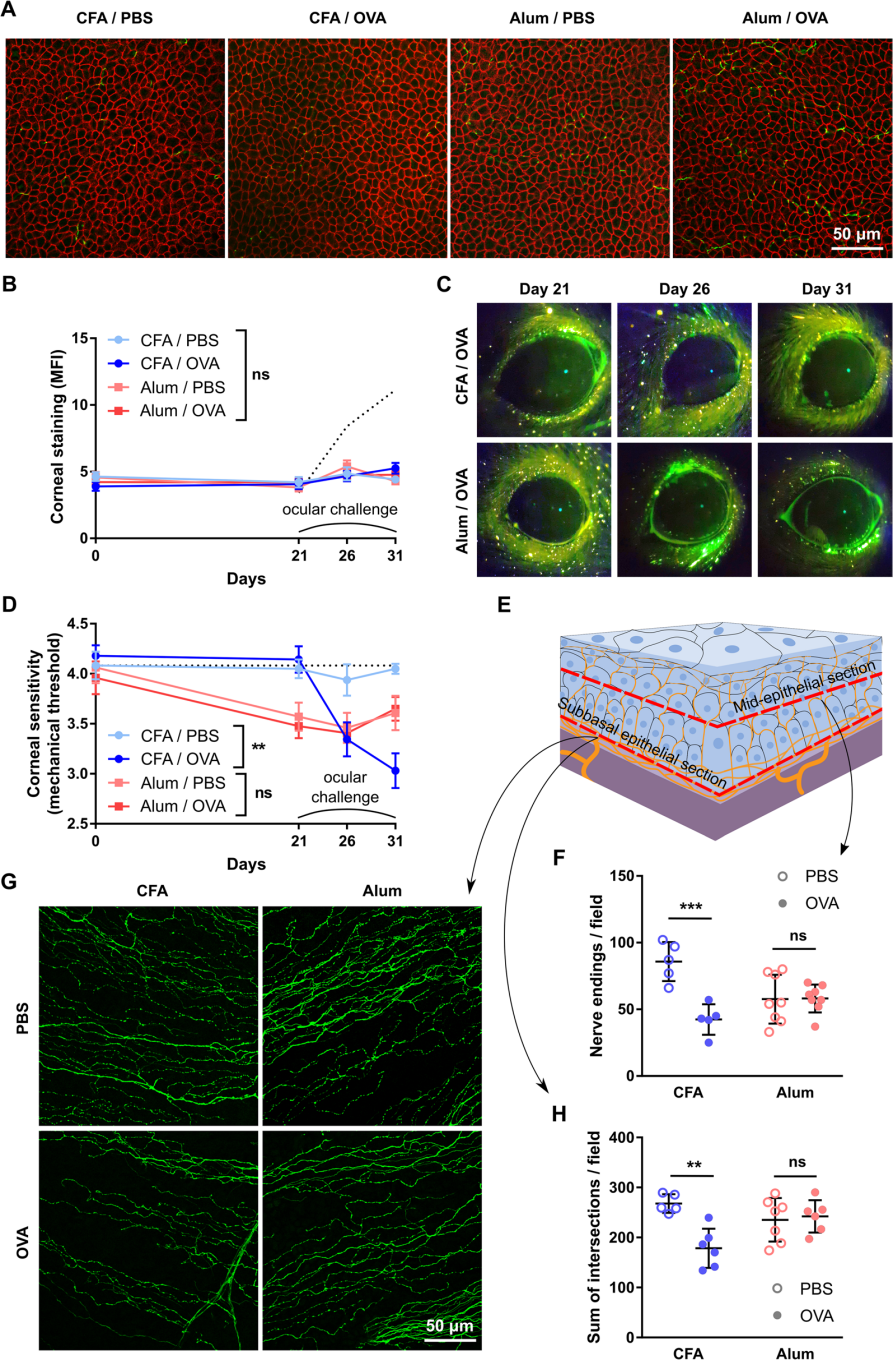
Effect of a local Th1- and Th2-skewed immune response on the corneal epithelium and nerves of transgenic CD4^+^ T cell receptor mice. OT-II mice were immunized with ovalbumin (OVA) in combination with complete Freund’s adjuvant (CFA) or alum and later given OVA or saline (PBS) eye drops, as detailed in the previous figure. **A** Representative micrographs of corneal whole mounts stained with E-cadherin (red) and tubulin β3 (green) from immunized OT-II mice. **B** Cumulative data and **C** representative micrographs of corneal dextran-fluorescein uptake in immunized OT-II mice. The dotted reference line corresponds to wild-type C57/BL6 mice with dry eye surgically induced on the same day as the ocular challenge (positive control for corneal epitheliopathy). **D** Corneal mechanical sensitivity thresholds of immunized mice over the 31-day experiment. The dotted reference line corresponds to the average baseline measurements of all the mice in the experiment. **E** Schematic of the levels at which corneal nerve morphology was analyzed. **F** Density of vertical intraepithelial nerve terminals in mid-epithelial corneal sections (representative example). **G** Representative micrographs of mid-peripheral subbasal sections (tubulin β3 staining) and **H** pooled data (representative experiment) of corneal neural complexity quantification (sum of intersections, Sholl analysis). All experiments were performed twice or more with 6 mice/group/experiment. For all experiments, mean ± standard error of measurement is shown. Two-way ANOVA (immunization and ocular challenge) with Sidak’s post hoc test was used to compare means. *p < 0.05, **p < 0.01, and *ns* not significant

Once we had determined that the immunization protocols induced the expected Th1/Th2 skewing, we examined the resulting ocular surface immune responses. Three weeks after immunization, both Th1- and Th2-skewed mice were challenged with daily OVA eye drops for 10 days to elicit a local adaptive immune response. In both Th1- and Th2-skewed mice, daily antigenic challenge led to ocular inflammatory changes (day 31, Fig. 1G) while saline instillation had no appreciable effect (data not shown). Antigenic challenge also increased the number of CD4^+^ T cells in the conjunctiva, indicative of local recruitment and activation of these cells (day 31, Fig. 1H). After we had established that both models had comparable activation of antigen-specific ocular surface immune responses with either Th1- or Th2-skewing, we assessed the impact of said immune responses on the corneal epithelium and corneal nerves. In both cases, there were no changes in the morphology of epithelial cells or the integrity of intercellular junctions after 10 days of local immune activation (day 31, Fig. 2A). In addition, we observed no increase in the uptake of a fluorescent dye applied topically to the corneal surface, a clinically validated indicator of corneal epithelial barrier integrity (Fig. 2B, C). Comparable measurements taken from wild-type mice with dry eye are provided as reference (positive control for corneal epitheliopathy development, dotted line starting on day 21 in Fig. 2B). These findings indicate that the activation of CD4^+^ T cells in the ocular surface did not appreciably damage the corneal epithelium.

By contrast, we observed significant changes in the corneal nerves in both Th1- and Th2-skewed models. Regarding nerve function, we measured corneal mechanical sensitivity using a mouse-adapted modification of the Cochet-Bonnet esthesiometry technique employed in the clinic [7, 34]. Corneal mechanical sensitivity in saline-challenged Th1-skewed mice remained unchanged throughout the experiment (Fig. 2D). Remarkably, OVA-challenged Th1-skewed mice showed a significant decline in corneal mechanical sensitivity after five days of antigenic challenge (day 26) that progressively worsened over the 10 days through 10 days of ocular OVA exposure (day 31). By contrast, Th2-skewed mice exhibited a significant drop in corneal mechanical sensitivity already after 5 days of alum administration (and more than two weeks before ocular antigenic challenge). Moreover, this decline did not worsen by ocular OVA instillation in Th2-skewed mice (Fig. 2D). The fact that corneal nerve dysfunction in Th2-skewed mice was independent of topical antigenic challenge (and thus of local activation of T cells) and that it was already detectable after 5 days of immunization (data not shown) suggested that it was related to aluminum neurotoxicity [51]. Confocal microscopy analysis of nerve morphology in corneal whole mounts (Fig. 2E) was concordant with the data on corneal nerve function. OVA-challenged Th1-skewed mice showed a significantly lower density of intraepithelial nerve terminals (Fig. 2F) and reduced complexity of the intraepithelial basal nerve plexus (Fig. 2G, H), whereas saline-challenged Th1-skewed mice had comparable corneal nerve morphology to naïve mice. By contrast, both saline- and OVA-challenged Th2-skewed groups exhibited altered nerve morphology but the changes were less marked than in OVA-challenged Th1-skewed mice (Fig. 2E–G). Altogether these findings suggest that although alum administration does seem to have a systemic effect on corneal nerves, only the activation of a Th1-skewed adaptive immune response at the ocular surface leads to the development of corneal neuropathy.

### A Th1-skewed immune response at the ocular surface also induces corneal neuropathy in mice with a wild-type T cell repertoire

**Fig. 3:**
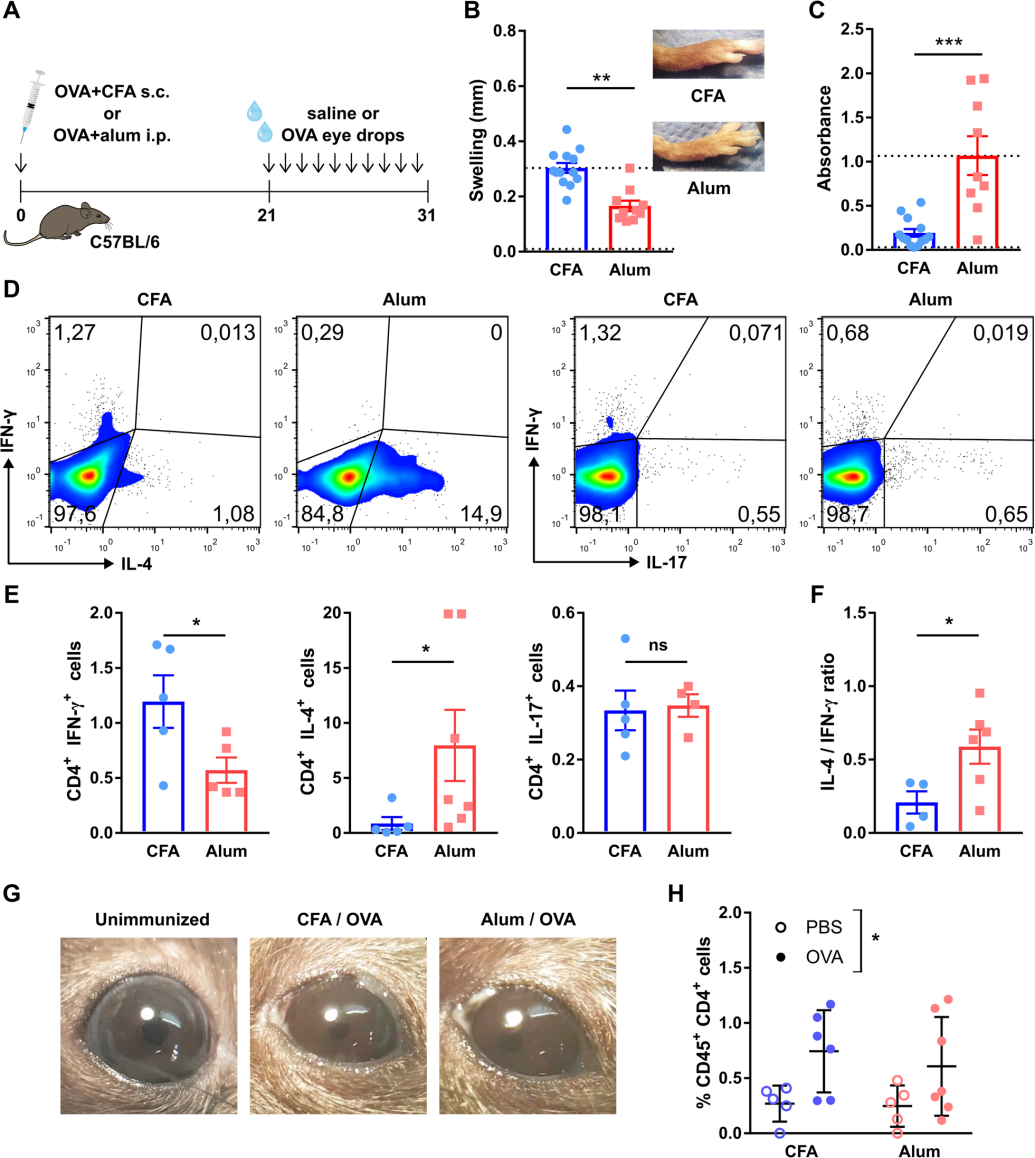
Th1 and Th2-skewing of the adaptive immune response in the ocular surface of wild-type mice. **A** Wild-type (wt) mice were immunized with OVA in combination with either complete Freund’s adjuvant (CFA) or alum to induce a Th1- or Th2-skewed immune response, respectively. Three weeks later, mice were given saline or OVA eye drops daily for 10 days to induce an ocular immune response. **B** Delayed-type hypersensitivity response to footpad OVA injection in immunized wt mice. Pooled data (left) and representative images (right) of footpads. **C** Serum OVA-specific IgE levels in wt mice 30 days after immunization as assessed by ELISA. Upper and lower reference lines correspond to positive (alum-immunized) and negative (non-immune) wild-type controls from another experiment. **D** Representative dot plots and **E** pooled data of interferon-γ (IFN-γ), interleukin (IL)-4, and IL-17 production by CD4^+^ T cells obtained from spleens of immunized mice. **F** Pooled data of ratio of IL-4/IFN-γ producing CD4^+^ T cells. **G** Representative photographs of mouse eyes on day 31 of the experiment. H) Conjunctival CD4^+^ T cells in immunized mice as assessed by flow cytometry (pooled data). All experiments were performed twice or more with 6 mice/group/experiment. For all experiments, mean ± standard error of measurement is shown. To compare means, Student’s t test was used for **B**, **C**, **E**, and **F**, and two-way ANOVA (immunization and ocular challenge) was used for **H**. *p < 0.05, **p < 0.01, ***p < 0.001, and *ns* not significant

Although mice transgenic for a T cell receptor are an invaluable tool for exploring T cell responses in vivo, their immune system exhibits certain abnormalities related to the large pool of CD4^+^ T cells that recognize the same antigen [45]. Because of this limitation, we validated the previous findings using wild-type C57BL/6 mice, which have a normal, highly diverse T cell repertoire that includes only a few OVA-specific CD4^+^ T cells. Upon OVA immunization in the presence of one of the two adjuvants (Fig. 3A), wild-type mice developed either a strong delayed-type hypersensitivity response to OVA (Fig. 3B), evidence of Th1-skewing, or high serum levels of OVA-specific IgE (Fig. 3C) indicative of Th2 polarization [46, 47]. As expected, CD4^+^ T cells from CFA-immunized mice produced higher levels of the signature Th1 cytokine IFN-γ whereas those from alum-immunized mice released more IL-4, the signature Th2 cytokine IL-4 (Fig. 3D). As was the case with T cell receptor transgenic OT-II mice, there was no statistically significant difference in IL-17 production, and the IL-4/IFN-γ ratio clearly showed the opposite immune skewing in the two groups (Fig. 3F). In both groups, daily antigenic challenge led to ocular inflammatory changes (Fig. 3G). Antigenic challenge also caused a comparable increase in conjunctival CD4^+^ T cells in both groups, indicative of local recruitment and activation of these cells (Fig. 3H).

**Fig. 4:**
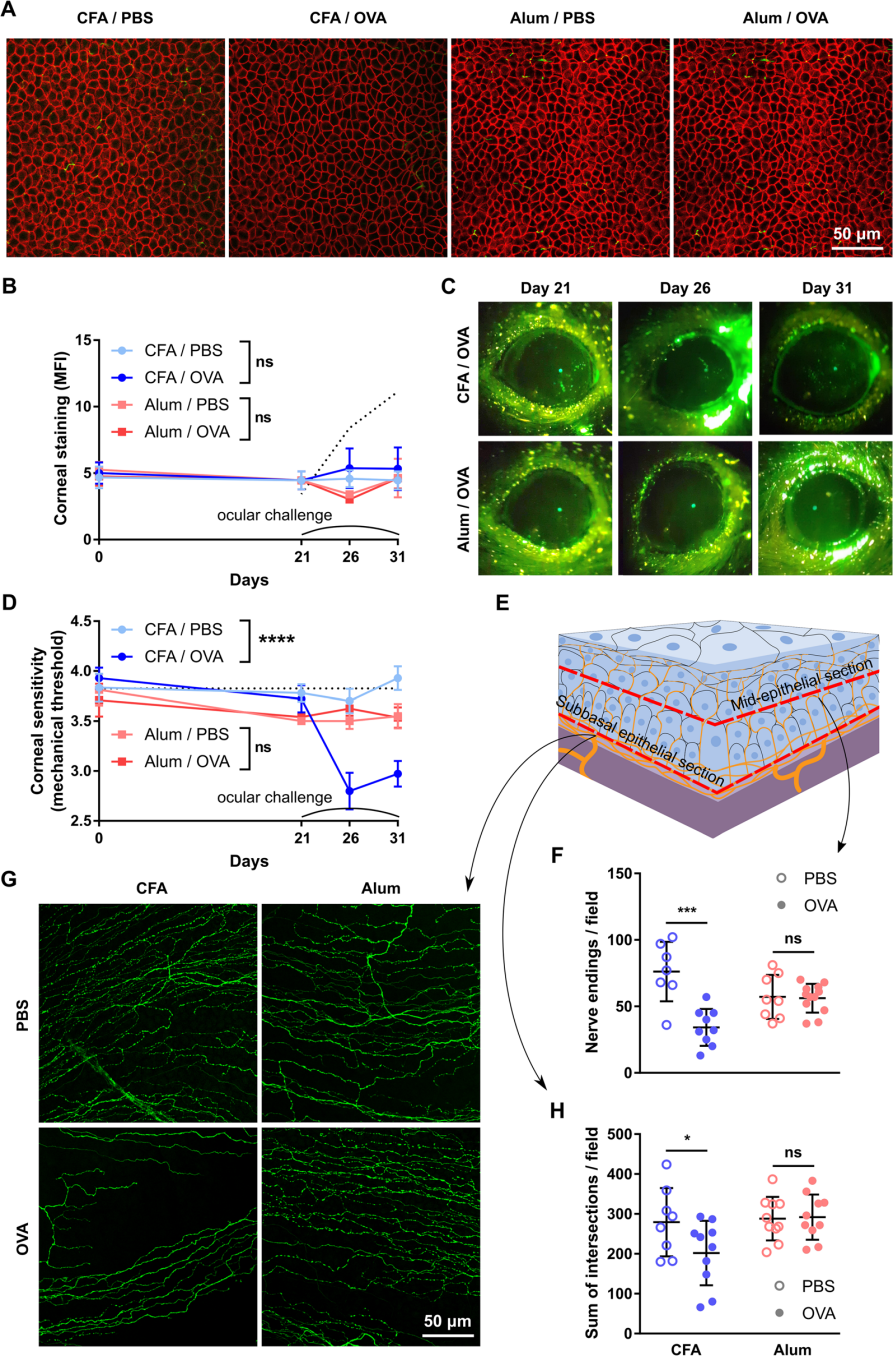
Effect of a local Th1- and Th2-skewed immune response on the corneal epithelium and nerves of wild-type mice. Wild-type (wt) mice were immunized with ovalbumin (OVA) in combination with complete Freund’s adjuvant (CFA) or alum and later given OVA or saline (PBS) eye drops, as detailed in the previous figure. **A** Representative micrographs of corneal whole mounts stained with E-cadherin (red) and tubulin β3 (green) from immunized wt mice. **B** Cumulative data and **C** representative of corneal dextran-fluorescein uptake in immunized wt mice. The dotted reference line corresponds to wt mice with dry eye surgically induced on the same day as the ocular challenge (positive control for corneal epitheliopathy). **D** Corneal mechanical sensitivity thresholds of immunized mice over the 31-day experiment. The dotted reference line corresponds to the average baseline measurements of all the mice in the experiment. **E** Schematic of the levels at which corneal nerve morphology was analyzed. **F** Density of vertical intraepithelial nerve terminals in mid-epithelial corneal sections (representative example). **G** Representative micrographs of mid-peripheral subbasal sections (tubulin β3 staining) and **H** pooled data (representative experiment) of corneal neural complexity quantification (sum of intersections, Sholl analysis). All experiments were performed twice or more with 6 mice/group/experiment. For all experiments, mean ± standard error of measurement is shown. To compare means, two-way ANOVA (immunization and ocular challenge) was used. *p < 0.05, **p < 0.01, ***p < 0.001, and *ns* not significant

Confirming the findings in T cell receptor transgenic mice, ocular challenge with OVA in wild-type C57BL/6 mice did neither induce morphological changes in corneal epithelial intercellular junctions (Fig. 4A) nor affect corneal barrier integrity (Fig. 4B, C). In line with our previous findings, OVA challenge in Th1-skewed C57BL/6 mice led to a progressive drop in corneal mechanical sensitivity while saline-challenged Th1-skewed remained unaffected. In Th2-skewed mice, alum induced a rapid decrease in corneal mechanical sensitivity that was detectable (day 5, data not shown) well before ocular challenge with either saline or OVA. Thus, alum-immunized wild-type mice also exhibited signs of systemic aluminum neurotoxicity (Fig. 4D). Confocal microscopy (Fig. 4E) showed that ocular OVA challenge in Th1-skewed mice significantly reduced the density of intraepithelial nerve terminals (Fig. 4F) and the complexity of the intraepithelial basal nerve plexus (Fig. 4G, H). Saline-challenged Th1-skewed mice had normal corneal nerve morphology. By contrast, both saline- and OVA-challenged Th2-skewed groups exhibited altered corneal nerve morphology but the changes were less marked than in OVA-challenged Th1-skewed mice (Fig. 4F–H).

These experiments ruled out potential artifacts introduced by the abnormally large number of antigen-responding T cells in OT-II mice because the observed changes in corneal nerve function and morphology were commensurate to those in wild-type mice. Moreover, the wild-type T cell repertoire allowed for better assessment of the Th1/Th2-skewing in this model. Thus, our findings in wild-type C57BL/6 mice were consistent with those obtained with T cell receptor-transgenic mice: only a Th1-skewed immune response at the ocular surface leads to corneal neuropathy development. Intriguingly, alum immunization per se was also associated with corneal nerve changes in wild-type mice, indicating the presence of an adjuvant effect that required additional testing.

### CD4^+^ T cells from Th1-skewed mice but not from Th2-skewed mice promote corneal neuropathy

**Fig. 5:**
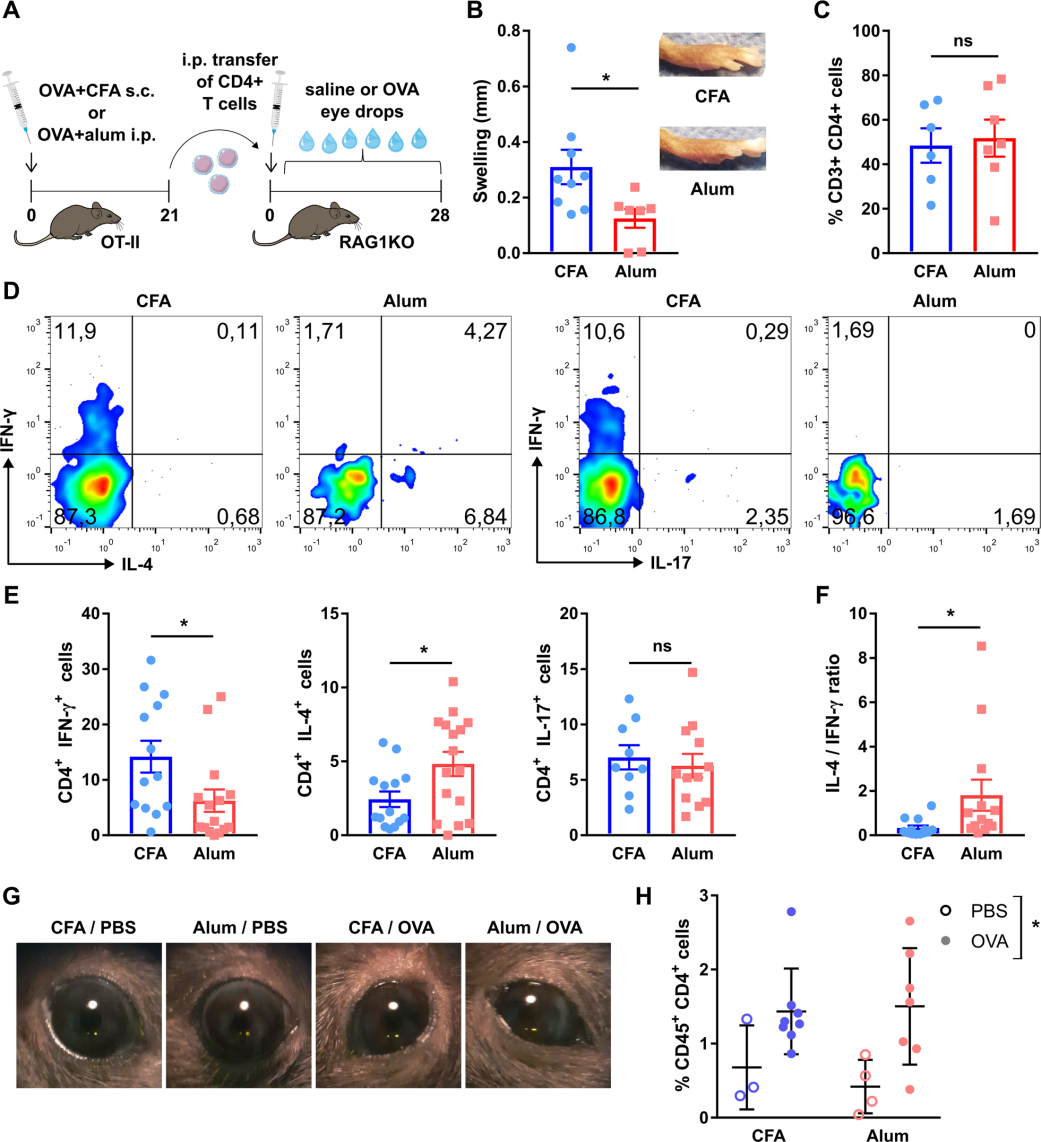
Adoptive transfer of ex vivo Th1 or Th2-skewed CD4^+^ T cells in T cell-deficient mice. **A** OT-II mice [transgenic for an ovalbumin (OVA)-specific MHC II-restricted T cell receptor) were immunized with OVA in combination with either complete Freund’s adjuvant (CFA) or alum to induce a Th1- or Th2-skewed immune response, respectively. Three weeks later, their splenic and lymph node CD4^+^ T cells were isolated and transferred i.p. to recombination-activating gene (RAG)-1 knockout mice (T cell-deficient, 1 × 10^6^ cells/mouse) that were given saline or OVA eye drops daily for 4 weeks to induce an ocular immune response. **B** Delayed-type hypersensitivity response to footpad OVA injection in transferred mice. Pooled data (left) and representative images (right) of footpads. **C** Proportion of CD4^+^ T cells in cervical lymph nodes of adoptively transferred mice (representative experiment). **D** Representative dot plots and **E** pooled data of interferon-γ (IFN-γ), interleukin (IL)-4, and IL-17 production by CD4^+^ T cells obtained from lymph nodes of transferred mice. **F** Pooled data of ratio of IL-4/IFN-γ producing CD4^+^ T cells. **G** Representative photographs of transferred mouse eyes after 4 weeks of ocular challenge. **H** Conjunctival CD4^+^ T cells in transferred mice as assessed by flow cytometry (representative experiment). All experiments were performed twice or more with 6 mice/group/experiment. For all experiments, mean ± standard error of measurement is shown. To compare means, Student’s *t* test was used for **B**, **C**, **E**, and **F**, and two-way ANOVA (cell source and ocular challenge) was used for **H**. *p < 0.05, **p < 0.01, and *ns* not significant

Although the previous findings indicated that a Th1-skewed immune response is required for corneal neuropathy to develop, the use of different adjuvants did not allow for controlling the magnitude of the resulting immune response, which could have been stronger in one group. In addition, alum-based immunization in and of itself affected corneal nerves in the Th2-skewed group. To account for these potential confounders, we focused on CD4^+^ T cells because they orchestrate the adaptive immune response. We combined the previous protocol with an adoptive transfer setup, which allowed us to isolate the cells of interest from all other immune cells and to limit the neurotoxic effect of alum while controlling for the number of CD4^+^ T cells. First, we immunized OT-II mice to generate a large number of Th1- or Th2-biased antigen-specific CD4^+^ T cells, as we had already established that the ocular surface findings using this transgenic T cell receptor model could be replicated in wild-type mice (Figs. 3, 4). Three weeks later, we isolated the CD4^+^ T cells from the spleen and lymph nodes of either Th1- or Th2-skewed mice, which were adoptively transferred into recombination activating gene 1 (RAG1)-deficient mice lacking T and B cells. Both groups of RAG1^−/−^ recipient mice were challenged daily for 4 weeks with either saline or OVA eye drops to activate the transferred cells at the ocular surface (Fig. 5A). We confirmed the successful transfer of a Th1-skewed OVA-specific immune response by observing a strong DTH response only in the Th1-reconstituted mice (Fig. 5B). Due to the lack of antibody-producing B cells in RAG1^−/−^ mice, OVA-specific serum IgE could not be used as an indicator of Th2 reconstitution. Then, as CD4^+^ T cells expand vigorously upon transfer into a lymphopenic host [52], we verified that the extent of immune reconstitution was comparable between groups. As shown in Fig. 5C, there was no statistically significant difference in the proportion of CD4^+^ T cells in the lymph nodes of Th1- and Th2-transferred mice. Also, ocular OVA instillation similarly increased the total number of recovered cells from the cervical lymph nodes in both Th1- and Th2-reconstituted mice (2.8- vs 2.4-fold, respectively), consistent with local presentation of the antigen by eye-derived antigen-presenting cells and subsequent CD4^+^ T cell activation and proliferation. As effector CD4^+^ T cells exhibit plasticity and may switch to another differentiation profile in vivo [53], we verified by flow cytometry that the transferred CD4^+^ T cells in the reconstituted mice retained their original profile after 4 weeks of proliferation and recirculation in vivo. CD4^+^ T cells from Th1-recipient mice produced more IFN-γ and less IL-4 than those from Th2-recipient mice while there was no difference in the proportion of IL-17+ CD4^+^ T cells (Fig. 5D, E). As for the previous experiments, the IL-4/IFN-γ ratio confirmed the skewing of the CD4^+^ T cell compartment in the two mouse groups (Fig. 5F). Of note, the OT-II CD4^+^ T cells were no longer restricted by the immune regulatory signals from their immunocompetent hosts once transferred into immunodeficient RAG1^−/−^ recipient mice, and therefore their phenotype was amplified. Thus, the observed cytokine production and the corresponding Th1/Th2 polarization were consistent but more robust than in the original setup (Fig. 1). Finally, both Th1- and Th2-transferred mice developed comparable signs of ocular inflammation after antigenic challenge (Fig. 5G) and did not differ in the number of conjunctival CD4^+^ T cells (Fig. 5H), indicating comparable recruitment of antigen-specific CD4^+^ T cells to the ocular surface. Altogether these findings validated the model as a means to compare the effect of CD4^+^ T cell activation on the ocular surface.

**Fig. 6:**
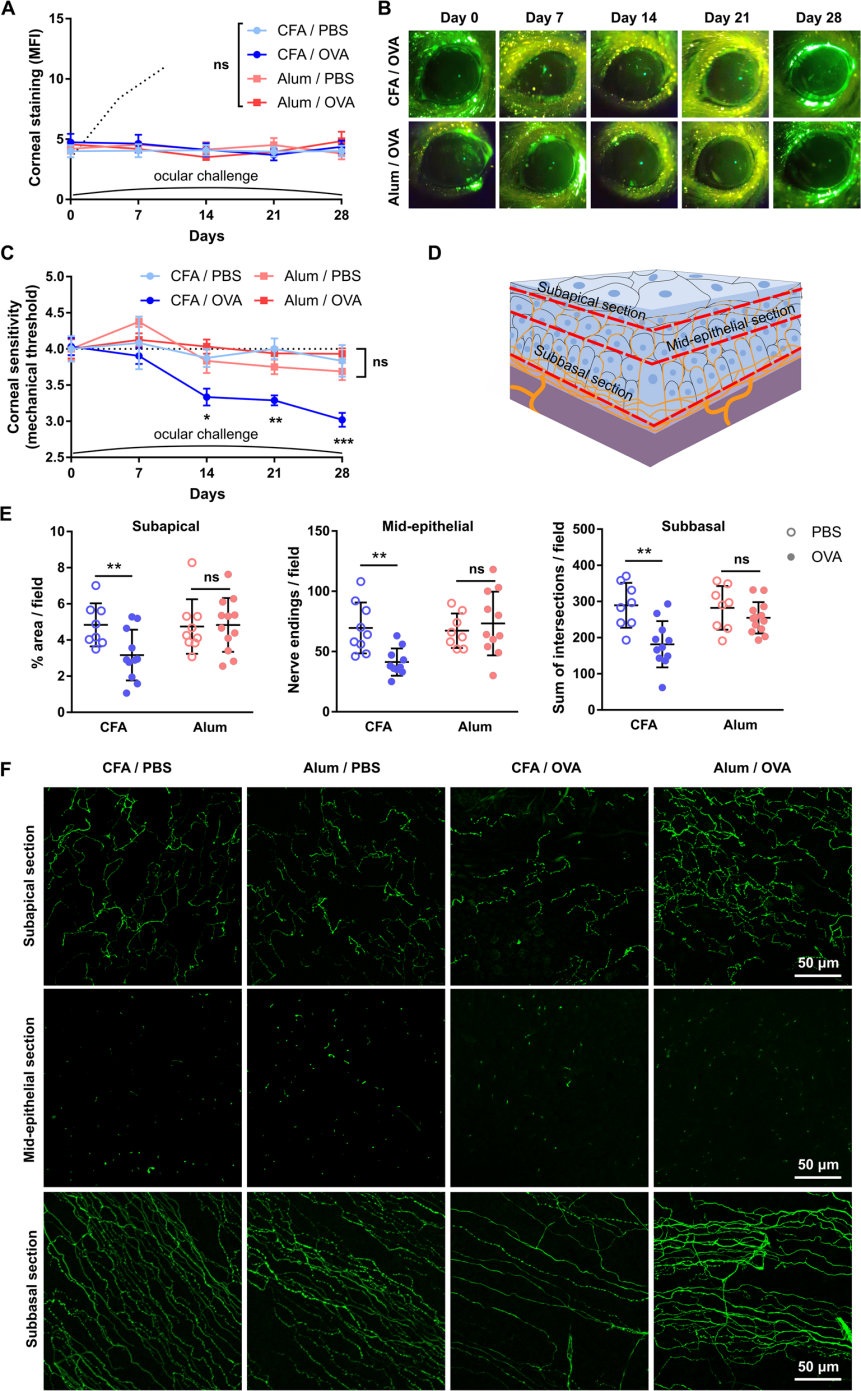
Effect of a local Th1- and Th2-skewed immune response on the corneal epithelium and nerves of CD4^+^ T cell-reconstituted mice. T cell-deficient mice were reconstituted with CD4^+^ T cells from mice immunized with ovalbumin (OVA) in combination with complete Freund’s adjuvant (CFA) or alum. Transferred mice were later given OVA or saline (PBS) eye drops daily for 4 weeks as detailed in the previous figure. **A** Representative micrographs and **B** pooled data of corneal dextran-fluorescein uptake in reconstituted mice challenged with ocular OVA. **C** Corneal mechanical sensitivity thresholds of reconstituted mice given either PBS or OVA eye drops over 4 weeks. The dotted reference line corresponds to the average baseline measurements of all the mice in the experiment. **D** Schematic of the levels at which nerve morphology was analyzed in corneal whole mounts stained with tubulin β3 (green). **E** Quantification (cumulative data) of intraepithelial nerve terminals imaged *en face* beneath the apical epithelial squamous cells (subapical section) and in cross-section at the mid-epithelial level (count of nerve endings/field) as they run perpendicularly to the surface, and of corneal neural complexity at the subbasal level (sum of intersections, Sholl analysis). **F** Representative micrographs of corneal intraepithelial nerves at the three different levels. All experiments were performed twice or more with 6 mice/group/experiment. For all experiments, mean ± standard error of measurement is shown. To compare means, two-way ANOVA was used in **A** and **C** (treatment and time), and **E** (immunization and challenge). *p < 0.05, **p < 0.01, ***p < 0.001, and *ns* not significant

In line with the previous experiments, neither group of reconstituted mice showed signs of impaired corneal barrier function over 4 weeks of daily saline or OVA instillation (Fig. 6A, B). This finding indicates that neither Th1-skewed nor Th2-skewed CD4^+^ T cells drive detectable changes in the corneal epithelium when activated in the absence of additional stressors. By contrast, we observed changes in corneal nerve function and morphology but only in antigen-challenged Th1-transferred mice. Activation of CD4+ T cells at the ocular surface decreased corneal mechanical sensitivity in the Th1-transferred group while having no effect in Th2-transferred mice (Fig. 6C). Consistently, there was a significant change in the morphology of intraepithelial corneal nerves only in OVA-challenged Th1-transferred mice (Fig. 6D): a decrease in the area occupied by subapical nerve endings, the density of vertical nerve terminals, and the complexity of the subbasal nerves (Fig. 6E, F). Altogether these experiments excluded the previously observed neurotoxic effect of alum (Figs. 1, 2, 3, 4), allowing us to confirm that Th1-skewed but not Th2-skewed CD4^+^ T cells are instrumental in driving corneal neuropathy when activated at the ocular surface.

### In vitro polarized Th1 CD4^+^ T cells but not Th2 or Th17 T cells promote corneal neuropathy

**Fig. 7:**
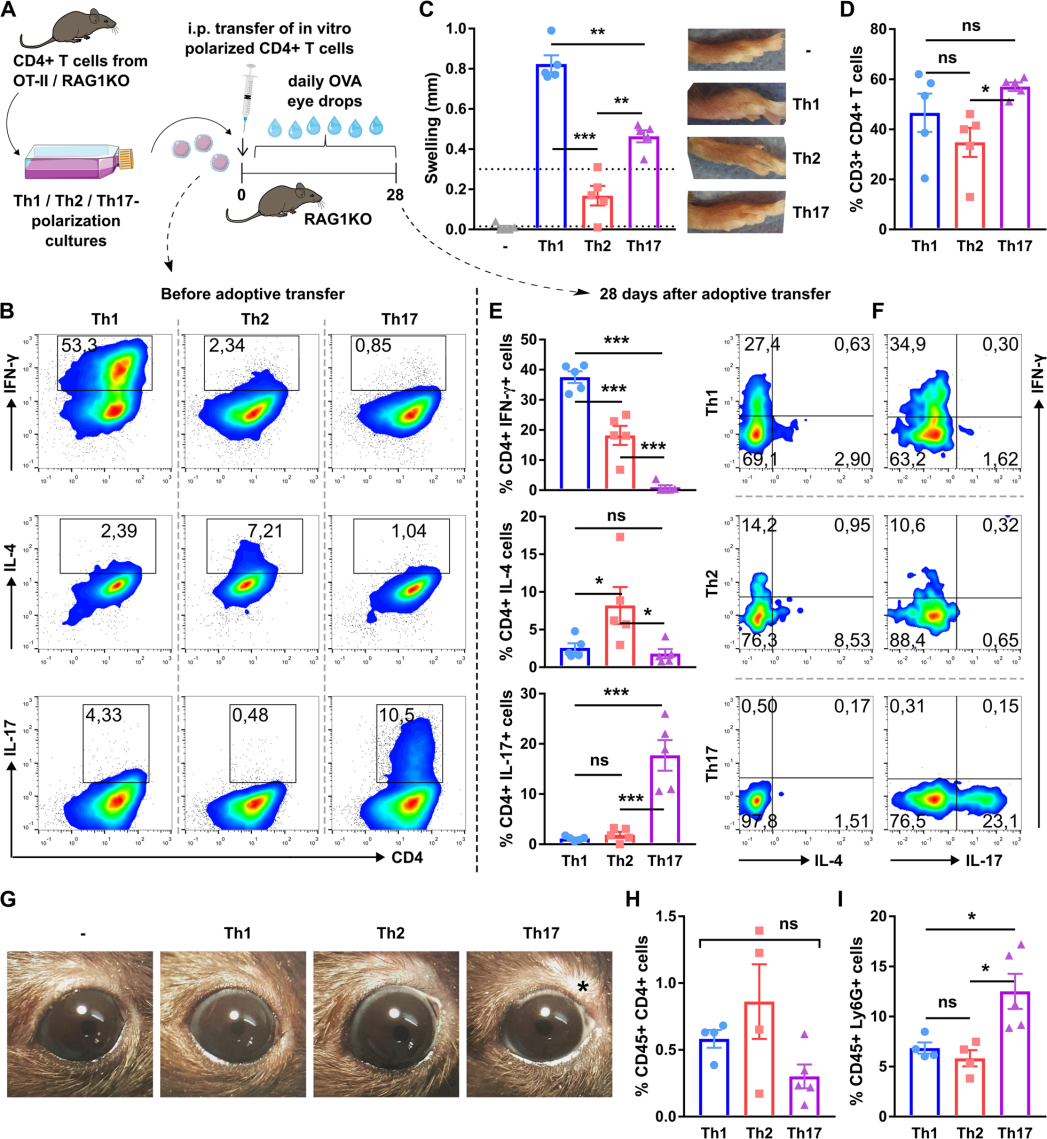
Adoptive transfer of in vitro polarized Th1, Th2, or Th17 CD4^+^ T cells to T cell-deficient mice. **A** CD4^+^ T cells were isolated from the spleen and lymph nodes of OT-II [transgenic for an ovalbumin (OVA)-specific MHC II-restricted T cell receptor)]/recombination-activating gene 1 (RAG1)-deficient mice and cultured under Th1, Th2, and Th17-promoting conditions for 5 days. The resulting polarized cells were transferred i.p. to RAG1-deficient mice (T cell-deficient, 1 × 10^6^ cells/mouse) that were given OVA eye drops daily for 4 weeks to induce an ocular immune response. A group of non-transferred (–) littermates was also included. **B** Representative dot plots of interferon-γ (IFN-γ), interleukin (IL)-4, and IL-17 production by CD4^+^ T cells after 5 days of in vitro polarization and before adoptive transfer. **C** Delayed-type hypersensitivity response to footpad OVA injection in recipient mice. Representative experiment (left) and images (right) of footpads. **D** Proportion of CD4^+^ T cells in the cervical lymph nodes of adoptively transferred mice (representative experiment). **E** Representative dot plots and **F** pooled data of IFN-γ, IL-4, and IL-17 production by CD4^+^ T cells obtained from cervical lymph nodes of mice 28 days after the adoptive transfer. **G** Representative photographs of transferred mouse eyes after 4 weeks of ocular challenge. * indicates a psoriasiform lesion. **H** Conjunctival CD4^+^ T cells and **I** neutrophils in transferred mice as assessed by flow cytometry (representative experiment). All experiments were performed twice or more with 5 mice/group/experiment. For all experiments, mean ± standard error of measurement is shown. To compare means, one-way ANOVA with post hoc testing was used. *p < 0.05, **p < 0.01, ***p < 0.001, and *ns* not significant

Adjuvants potentiate and polarize the immune response, resulting in a CD4^+^ T subset (Th1/2/17) that predominates in the T cell expansion while coexisting with others [54, 55]. Thus, CD4^+^ T cells isolated from either CFA or alum-immunized mice are mostly Th1 or Th2, respectively, but some Th17 cells can be detected as well (Figs. 1, 3, and 5). To better control T cell polarization, we resorted to the in vitro differentiation of either Th1, Th2, or Th17 CD4^+^ T cells and their subsequent adoptive transfer to RAG1-deficient mice (Fig. 7A), as for the previous experiment (Fig. 5A). To this aim, we isolated CD4^+^ T cells from OT-II/RAG1^−/−^ mice, which only express OVA-specific T cell receptors on their CD4^+^ T cells and have no CD8^+^ T cells or B cells. These CD4^+^ T cells were cultured under three sets of well-characterized differentiation conditions (see Methods). As shown in Fig. 7B, the cells acquired strong polarization to either Th1, Th2, or Th17 profiles over 5 days of culture. Then, four groups of RAG1-deficient mice were reconstituted with one of the cultures or saline as a control. Of note, naïve CD4+ T cells were not included as a control due to the impossibility of controlling their in vivo polarization and its potentially confounding influence [52, 56]. We also simplified the experimental setup by excluding ocular saline instillation as a control since we had previously established that local antigen-specific activation of CD4^+^ T cells is required for immune-mediated corneal epitheliopathy to ensue in this model (Figs. 5, 6). Therefore, all the mice adoptively transferred with in vitro polarized CD4^+^ T cells received OVA eye drops daily for four weeks, and so did the control group of non-transferred mice (Fig. 7A). First, we verified the reconstitution with polarized CD4^+^ T cells in the transferred mice by measuring antigen-specific DTH reactions (Fig. 7C). As expected, non-transferred mice showed no reaction to OVA due to their lack of an adaptive immune response. By contrast, Th1-reconstituted mice displayed local footpad swelling > twofold higher than historical wild-type controls immunized with OVA and a Th1-inducing adjuvant (CFA). Th17-reconstituted mice showed smaller but still strong swelling responses and Th2-reconstituted mice displayed the weakest responses. Reconstitution was comparable among the three groups, as assessed by the proportion of CD4^+^ T cells in the cervical lymph nodes (Fig. 7D). Th17-reconstituted mice had a slightly higher proportion of lymph node CD4^+^ T cells than Th2-reconstituted mice, which we attribute to different proliferation rates in vivo of each effector cell type. Of note, we also observed a similar trend during in vitro polarization, as Th17 cultures consistently yielded the highest number of cells while Th2 cultures yielded the lowest. Finally, as reconstitution depends on extensive in vivo proliferation of the transferred cells in a different environment from that in which they grew in vitro, we verified that the CD4^+^ T cells retained their original polarization after 4 weeks in vivo. As depicted in Fig. 7E and F, all three groups exhibited strong polarization consistent with the original profiles. With this evidence, we established that the experimental setup allowed for a fair comparison of the effect of highly polarized adaptive immune responses.

Regarding the ocular phenotype, OVA eye drop administration had no appreciable effect on control RAG1-deficient mice while it caused ocular surface inflammation (lid edema) in all three groups of reconstituted mice (Fig. 7G). Th2- and Th17-reconstituted mice had more conjunctival discharge. Of note, we observed periocular psoriasiform lesions in or around the lids of Th17-reconstituted mice, which we attribute to cutaneous activation of Th17 CD4^+^ T cells induced by periocular spilling of the OVA-containing eye drops [57]. Conjunctival recruitment of CD4^+^ T cells was not significantly different among the three groups (Fig. 7H) and in line with previous experiments (Figs. 1H, 3H, and 5H). Thus, despite the mild differences observed in the extent of reconstitution (Fig. 7D), the three groups were comparable in the frequency of ocular surface CD4^+^ T cells, which was within the physiological range observed in the previous experiments. We also confirmed the polarized nature of the immune response by analyzing the number of conjunctival neutrophils. Th17 CD4^+^ T cells are known to recruit neutrophils to mucosal surfaces [58], and in line with this, we observed an increase in conjunctival neutrophils only in Th17-transferred mice (Fig. 7I).

**Fig. 8:**
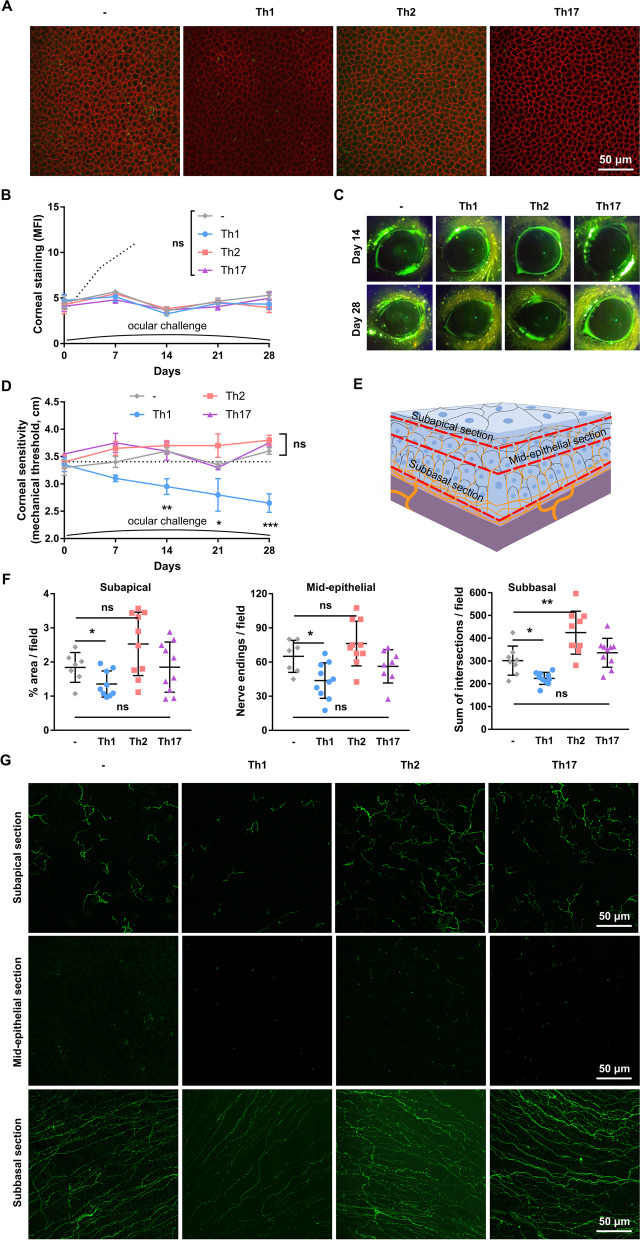
Effect of the local activation of highly polarized Th1, Th2, and Th17 CD4^+^ T cells on the corneal epithelium and nerves of T cell-reconstituted mice. T cell-deficient mice were reconstituted with in vitro polarized CD4^+^ T cells. Transferred mice were later given OVA or saline (PBS) eye drops daily for 4 weeks as detailed in the previous figure. A group of non-transferred (–) littermates was also included. **A** Representative micrographs of corneal whole mounts stained with E-cadherin (red) and tubulin β3 (green) from immunized wt mice. **B** Representative micrographs and **C** pooled data of corneal dextran-fluorescein uptake in transferred mice. **D** Corneal mechanical sensitivity thresholds of reconstituted mice given OVA eye drops over 4 weeks. The dotted reference line corresponds to the average baseline measurements of all the mice in the experiment. **E** Schematic of the levels at which nerve morphology was analyzed in corneal whole mounts stained with tubulin β3 (green). **F** Quantification (cumulative data) of intraepithelial nerve terminals imaged en face beneath the apical epithelial squamous cells (subapical section) and in cross-section at the mid-epithelial level (count of nerve endings/field) as they run perpendicularly to the surface, and of corneal neural complexity at the subbasal level (sum of intersections, Sholl analysis). **G** Representative micrographs of corneal intraepithelial nerves at the three different levels. All experiments were performed twice or more with 6 mice/group/experiment. For all experiments, mean ± standard error of measurement is shown. To compare means, two-way ANOVA was used in **B** and **C** (treatment and time) and one-way ANOVA with Dunnett's post hoc test was used in (**F**). *p < 0.05, **p < 0.01, ***p < 0.001, and *ns* not significant

As was the case with polarized CD4^+^ T cells obtained ex vivo, the transfer of in vitro polarized CD4^+^ T cells neither appreciably changed corneal epithelial morphology (Fig. 8A) nor modified the corneal uptake of fluorescent dye over the 4-week experiment (Fig. 8B, C). These findings confirm that the isolated activation of CD4^+^ T cells of either effector profile is not sufficient to cause corneal epitheliopathy. By contrast, signs of corneal neuropathy (reduced corneal mechanical sensitivity) only developed in Th1-transferred mice (Fig. 8D). This finding was in agreement with the previous experiments, although the tempo of corneal neuropathy was faster in this setup: Th1-reconstituted mice exhibited significantly decreased corneal mechanical sensitivity already after one week of adoptive transfer. Of note, the transfer of Th17 cells did not cause corneal hypoesthesia, ruling out a possible pathogenic effect of the accompanying Th17 CD4^+^ T cells in the previous experiments. Corneal nerve morphology reflected the changes observed in corneal sensitivity measurements (Fig. 8E–G). Compared with non-transferred mice, Th1-transferred mice had smaller subapical nerve endings and decreased density of mid-epithelial nerve terminals (Fig. 8F) whereas Th2- and Th17-transferred mice did not experience significant changes. Regarding the subbasal nerve plexus (Fig. 8F), Th1-transferred mice also exhibited decreased complexity at this level. Of note, Th2-transferred mice had markedly increased complexity; by contrast, Th17-transferred mice did not show significant changes compared to non-transferred mice. Altogether, the data show that ocular activation of Th1 CD4^+^ T cells leads to impaired corneal function and altered nerve morphology. These findings confirm the predominating contribution of Th1 CD4^+^ T cells to immune-driven corneal nerve damage independently of corneal epitheliopathy.

## Discussion

Many ocular surface disorders have an immune pathophysiology. Corneal nerve dysfunction and morphological abnormalities are frequent findings in ocular surface disease, yet their pathogenesis is incompletely understood. Here we show that the development of a type 1-predominant local immune response leads to the onset of corneal neuropathy in the absence of other noxious agents that can trigger innate immunity or cause tissue injury, such as desiccation or microbial infections. We also show that among the diverse components of type 1 immune responses, Th1 CD4^+^ T cells actively promote corneal nerve damage. Of note, corneal neuropathy was observed in the absence of overt corneal epithelial pathology, indicating that corneal nerves are directly affected by the immune response and not as a consequence of corneal epitheliopathy. Thus, corneal nerves are more susceptible to purely type 1 immune-driven damage than corneal epithelial cells.

In this study, we determined the presence of corneal neuropathy by functional testing and morphological characterization of corneal nerve fibers. In terms of function, there are three well-defined types of nerve fibers in the cornea: mechanoreceptors, cold thermoreceptors, and polymodal nociceptors [3–5, 59]. We relied solely on mechanical sensitivity for assessing corneal nerve function for several motives. First, there is a highly reproducible method in widespread clinical and experimental use that shows correlation with morphological findings in animal models: Cochet-Bonnet esthesiometry and its mouse-adapted version [6, 7, 18, 34, 38, 60–62]. Second, adequate testing of cold thermoreceptors and polymodal nociceptors requires isolated electrophysiological measurements of single corneal fibers in the excised mouse eyes, which was incompatible with our experimental design [63]. Third, alternative methods such as quantification of blinking rates or nocifensive behavior in response to diverse agonists do not necessarily provide direct evidence of corneal neuropathy because the readouts are conditioned by numerous factors: the non-linear effect of axonal damage on fiber excitability [64–66], the influence of the environment and sex [67, 68], and the development of sensory crosstalk between cold thermoreceptors and polymodal nociceptors in the context of ocular surface inflammation [66]. In terms of nerve morphology, we analyzed the intraepithelial fibers at different levels (subapical, mid-epithelial, and subbasal) as they are the most affected segment of the corneal innervation in ocular surface disorders [5, 6]. Of note, comparable functional testing and morphological analysis criteria are currently being used to define the presence of corneal nerve damage in human clinical trials [69].

Our findings derive from four murine models of an antigen-driven ocular surface immune response, a form of adaptive immunity in which CD4^+^ T cells are at center stage. CD4^+^ T cells orchestrate the adaptive immune response by providing signals (activating and inhibitory cytokines) that recruit and potentiate cellular and humoral effectors [70]. Depending on the conditions under which they become activated, CD4^+^ T cells acquire different properties that fit within profiles or types of immune responses [70–72]. Thus, according to the prevailing paradigm, Th1 CD4^+^ T cells are the ones that predominantly produce IFN-γ, which potentiates the antimicrobial action of mononuclear phagocytes. Th1 CD4^+^ T cells are key elements of type 1 immune responses, which protect against intracellular pathogens. By contrast, Th2 CD4^+^ T cells produce IL-4, IL-5, and IL-13, which promote eosinophil and mast cell action and IgE production. Th2 CD4^+^ T cells coordinate type 2 immune responses against parasitic worms and toxins. Finally, Th17 CD4^+^ T cells participate in type 3 immune responses against extracellular bacteria and fungi by secreting IL-17 and IL-23, which activate mononuclear phagocytes, neutrophils, and epithelial cells [71]. The three types of effector immune responses have been described in ocular surface disorders, with the corresponding types of CD4^+^ T cells playing significant pathophysiological roles [8, 73]. For instance, Th1 CD4^+^ T cells induce a stress response in conjunctival goblet cells [74], while Th2 CD4^+^ T cells promote their survival and proliferation [75]. In ocular allergy, Th2 cell-derived cytokines increase corneal epithelial permeability [76], whereas Th17 CD4^+^ T cells disrupt the corneal epithelial barrier in dry eye [14, 17].

Regarding the contribution of the immune response to corneal nerve damage, a landmark study by Royer et al. showed that the development of sensory neuropathy in ocular surface disease is context-dependent and driven by complement and CD4^+^ T cells [18]. By comparing different murine models of ocular surface disorders, they found that CD4^+^ T cells promote corneal nerve damage in herpetic keratitis and ocular graft-versus-host disease but not in ocular allergy. Intriguingly, herpetic keratitis is an immune-mediated disease with Th1/Th17 predominance [77, 78], and IFN-γ levels in ocular GVHD increase as the cornea is infiltrated by donor T cells [79]. However, corneal levels of IL-6, a cytokine that favors Th17 CD4^+^ T cell differentiation, are also increased in both disorders [79, 80]. Thus, although the aforementioned findings hinted at a potential role of the predominant type of CD4^+^ T cells involved in each model (Th1/Th17 vs Th2), other factors could also explain the observed differences between the three models. One possibility is varying degrees of context-dependent, T cell-independent nerve damage. Herpes simplex virus-1 is neurotropic and invades corneal nerve fibers to gain access to trigeminal neuronal bodies, where it initiates latency [9]. Corneal nerve retraction in herpetic keratitis can occur in the absence of CD4^+^ T cells, although only transiently [81]. More recently, it was shown that in herpetic keratitis, both CD4^+^ T cells and myeloid cells secrete high levels of vascular endothelial growth factor-A, which is pathogenic to corneal nerves [82]. This body of evidence suggests that direct viral cytopathic effects or the initial inflammatory response to viral replication (mostly CD4^+^ T cell-independent) could also play a role in corneal nerve damage [80]. Another possibility is the context-dependent differences in the nature, location, and abundance of antigenic targets in herpetic keratitis, ocular GVHD, and ocular allergy. In herpetic keratitis, the specificity of CD4^+^ T cells is relevant because HSV1-infected mice that only harbor CD4^+^ T cells reactive against an irrelevant antigen do not develop sensory neuropathy [18]. In addition to pathogenic CD4^+^ T cells, corneal neuropathy onset requires an active corneal HSV1 infection, probably as a source of herpetic antigens expressed in corneal epithelial cells and nerves [18]. In line with this, allospecific CD4^+^ T cells that react against host-derived histocompatibility antigens expressed in corneal epithelial cells and nerves are at the heart of ocular graft-versus-host disease. By contrast, ocular allergy is driven by CD4^+^ T cells that react against a foreign antigen that is not expressed in the cornea but comes in contact with the ocular surface. Therefore, the circumstances under which ocular antigen presentation and T cell activation take place in each disorder are not comparable to draw a definitive conclusion about how the type of immune response affects corneal nerves.

By controlling the confounding factors mentioned above, our work sheds new mechanistic insight into the context-dependency of corneal sensory neuropathy. We observed that only Th1 CD4^+^ T cells promote corneal nerve damage in the absence of additional noxious stimuli and that this effect is independent of the development of corneal epithelial injury. Thus, we delineate the contribution of Th1 CD4^+^ T cells to purely immune-driven corneal neuropathy, as there are no other non-immune sources of inflammation or tissue damage in our models. We can also rule out bystander activation of CD4^+^ T cells in our models as local activation by ocular antigenic challenge was also required (Figs. 2, 4, saline-challenged Th1 mice did not develop corneal neuropathy). Nonetheless, this last assertion may not hold in a more physiological setting where other inflammatory signals may lower the threshold for CD4^+^ T cell activation. Royer and colleagues showed that corneal nerve damage relies on the activation of complement in the context of herpetic keratitis. Intriguingly, complement activation, through C3b deposition, also potentiates Th1 CD4^+^ T cell activation by CD46 signaling on the T cell membrane [54]. In another report, the adoptive transfer of CD4^+^ T cells from wild-type C57BL/6 mice with dry eye (enriched in Th1 cells) into RAG1-deficient mice induced corneal epithelial apoptosis by a mechanism that involves IFN-γ secretion at the ocular surface [13]. Although the impact on the corneal nerves was not explored in that report, its findings regarding corneal epitheliopathy induced by CD4^+^ T cells may at first seem in conflict with ours. However, it should be emphasized that the specificity of the transferred CD4^+^ T cells was quite different in both studies. In the report from Zhang et al. [13], the dry eye-derived CD4^+^ T cells were probably reacting to one or more corneal epithelial autoantigens whereas in our model they were exclusively specific for a non-corneal antigen. At any rate, our study shows that corneal nerves are more susceptible to purely type 1 immune-mediated damage than corneal epithelial cells.

Based on our adoptive transfer of highly polarized CD4^+^ T cells, it is tempting to speculate on the existence of an immune-driven epithelial–neural divide in corneal pathology. On the one hand, herein we demonstrate that corneal nerves are sensitive to type 1 but not to type 2 or type 3 immune responses that do not target corneal-specific antigens while corneal epithelial cells remain largely unaffected. On the other hand, the literature abounds in studies showing the pathogenic effect of CD4^+^ T cells on corneal epithelial cells in different immune contexts. In addition to the already discussed roles of Th1 and Th17 CD4^+^ T cells in dry eye and herpetic keratitis [8, 13, 14, 17, 73, 78], Th2 CD4^+^ T cells were shown to contribute to corneal epithelial barrier dysfunction in ocular allergy, a Th2-predominant immune disease in which corneal nerves are barely affected. They do so by secreting IL-9, which acts directly on IL-9 receptors in corneal epithelial cells and triggers changes in intercellular adhesion proteins [76]. Our findings complement the existing literature by providing evidence that the isolated activation of a Th1-predominant adaptive immune response is capable of inducing nerve pathology. However, additional pathogenic factors compound to the corneal changes observed in ocular surface disease. For instance, dry eye-associated ocular surface desiccation triggers an inflammatory program in corneal epithelial cells [83] that combined with innate immune activation [16] leads to the development of a Th1- and Th17-predominant adaptive immune response [8, 14]. In this pathogenic factor-rich context, Th1 and Th17 CD4+ T cells contribute to corneal epitheliopathy by several mechanisms [13, 14, 17, 84]. When interpreted in the disease context, our results suggest that the activation of Th1 CD4+ T cells in and of itself is sufficient to damage corneal nerves. By contrast, the same event or the corresponding activation of Th17 CD4+ in dry eye or Th2+ CD4+ T cells in the setting of ocular allergy promote corneal epitheliopathy in combination with other pathogenic factors (desiccation or allergen-specific effects, respectively). In line with this, we have previously shown that tear hyperosmolarity without desiccation causes immune disruption and corneal neuropathy but not overt corneal epitheliopathy in mice [85]. By contrast, desiccation poses a stronger environmental challenge to the ocular surface and leads to the full development of the three aspects of the disease in two different dry eye models [33, 86]. Our present findings provide further mechanistic insight to these observations. It remains to be established in our models if it is the activation of conjunctival CD4+ T cells that affects corneal nerves from a distance or if CD4+ T cells actually infiltrate the cornea and come in contact with corneal nerve fibers. The latter does occur in herpetic keratitis [9, 87, 88] and ocular GVHD [23], and intriguingly, tissue-resident memory T cells patrol the corneal epithelium after herpetic infection resolution in mice and in healthy humans [87].

There is also a sizable body of evidence supporting the roles of the different types of immune responses in the development of neuropathology beyond the ocular surface, mostly in the central nervous system [89]. In fact, the distinct phenotypes resulting from experimental manipulation of the immune response in murine models of autoimmune encephalomyelitis were instrumental in the discovery and characterization of Th17 CD4^+^ T cells in the first place [25, 26]. Less is known about the contribution of CD4^+^ T cells to peripheral nervous system pathology, and the focus is more on these cells shaping neuroregeneration after injury [24]. Intriguingly, neural injury models have shown that infiltrating Th1 CD4^+^ T cells foster Wallerian neurodegeneration while Th2 CD4^+^ T cells favor neuroregeneration through multiple effectors that include Schwann cells and macrophages [90]. Our findings are in line with these reports, as we provide strong evidence for a neurodegenerative effect of Th1 CD4^+^ T cells on corneal nerves. Conversely, there was a trend for a positive effect of Th2 CD4^+^ T cells on corneal nerve function and morphology but only in our adoptive transfer studies (Fig. 8). More recently, Th1 CD4^+^ T cells were shown to facilitate peripheral nerve inflammation by inducing macrophages to secrete pathogenic tumor necrosis factor-α [29]. However, it should be noted that all these models explore the fate of myelinated nervous tissue while most corneal nerves lose their myelin sheath after they cross the limbus to enter the stroma [3, 5]. Therefore, some observations derived from CD4^+^ T cells reactive to myelin-derived antigens may not apply to corneal neuropathology and more specific ocular surface studies are warranted.

Finally, the clinical relevance of our results is threefold. First, by showing that corneal nerves are directly affected by a Th1-predominant adaptive immune response we are contributing mechanistic insight to the pathogenesis of corneal neuropathy. As it was mentioned before, we did not model any disease in particular; our experiments represent the isolated activation of a local adaptive immune response. This event takes place in numerous ocular surface disorders such as herpetic keratitis, ocular graft-versus-host-disease, dry eye, and ocular allergy. Therefore, our findings contribute to the understanding of corneal neuropathy in such disease settings. Second, by showing that corneal neuropathy develops independently of corneal epitheliopathy and not as a consequence of corneal epithelial damage, we are potentially explaining why most treatment modalities in clinical use that were designed to improve corneal epithelial repair do not tackle this highly clinically relevant aspect of ocular surface disease. We need specific treatments for corneal neuropathy and these findings are rich in new directions for research. Third, our results show that the pathophysiology of corneal neuropathy has similarities with the pathophysiology of diverse forms of peripheral neuropathy where Th1 CD4+ T cells promote neural damage. Thus, some of the current knowledge and even therapeutic options for peripheral neuropathology may also apply to corneal neuropathology.

## Data Availability

Data sharing is not applicable to this article as no datasets were generated or analyzed during the current study.
